# A bridge between collinear inhibition and visual crowding: Hints from perceptual learning

**DOI:** 10.1177/03010066251360131

**Published:** 2025-07-29

**Authors:** Marcello Maniglia, Russell Cohen Hoffing

**Affiliations:** University of California, Riverside, Riverside, CA, USA; 1024 U.S. DEVCOM Army Research Laboratory, Adelphi, MD, USA

**Keywords:** collinear facilitation, visual crowding, perceptual learning, contrast sensitivity

## Abstract

Maniglia and colleagues reported a significant reduction in visual crowding following perceptual learning training on contrast detection using a lateral masking configuration with collinear flankers. They interpreted this reduction within a framework of shared cortical mechanisms between collinear inhibition, elicited by lateral masking with closely spaced flankers, and crowding. We reanalyzed their data to directly test this hypothesis by examining correlations between learning gains at short target-to-flankers separations (reduced contrast detection thresholds) and crowding reduction. Surprisingly, individual analyses revealed an inverse correlation: participants with greater reduction in collinear inhibition showed smaller reductions in crowding. We suggest that these participants exhibited separation-specific learning, which previous studies indicate may hinder effective transfer. Thus, while collinear inhibition and crowding may share mechanisms, distributed improvement across separations might be necessary to observe transfer of learning to crowding.

## Introduction

Collinear modulation and visual crowding are among the most studied phenomena in visual perception. In collinear modulation, the detection of a central target, usually a Gabor patch, is affected by the presence of iso-oriented flanking elements (Polat & Sagi, 1993): at short separation between the target and flankers, (<2λ in fovea,  < 6/8λ in periphery), contrast sensitivity decreases (collinear inhibition), while larger distances lead to an increase in sensitivity (collinear facilitation). In crowding, the identification of a central target, often a letter, is hindered by the presence of flanking elements placed within a so-called “critical space” (Levi, 2008).

While differing in methodology (collinear modulation is usually measured with oriented gratings, whereas crowding typically uses letters) and perceptual characteristics (collinear inhibition reduces detection, whereas crowding reduces discrimination), these two phenomena share several characteristics.

First, the strength of these effect seems to depend on the flanker separation: short distances are more deleterious for both crowding and lateral interaction, while larger separations reduce both.

Second, the inhibitory range of the flankers increases with eccentricity (Bouma, 1970; Lev & Polat, 2011; Maniglia et al., 2011, 2015a), possibly following cortical magnification. Levi and colleagues (Levi et al., 1984) suggested that crowding takes place when both target and flankers are placed within the same cortical “perceptual hypercolumn.” Similarly, Lev and Polat proposes that collinear modulation takes place between or within “perceptual fields” (Lev & Polat, 2011).

Third, the neural substrates of both phenomena, while still debated, are usually considered to be found in early visual cortex (Bringuier et al., 1999; Cass & Spehar, 2005; Gilbert & Wiesel, 1985, 1989; Grinvald et al., 1994; Levi & Carney, 2009; Pelli, 2008; Polat et al., 1998), hypothesis supported by animal electrophysiology and human neuroimaging studies(Henry & Kohn, 2022; Kapadia et al., 1995, 2000; Millin et al., 2014; Polat et al., 1998), with possibly feedback modulation from extra-striate areas (Chicherov et al., 2014; Levi, 2008; Maniglia et al., 2019; Whitney & Levi, 2011) As such, several authors have suggested that these phenomena might share, at least partially, the same neural substrates (Bonneh et al., 2007; Doron et al., 2015; Petrov et al., 2006; Polat & Sagi, 1993, 2007; Polat et al., 1997).

Fourth, learning studies show that training on collinear modulation transfers to reduction of crowding (Maniglia et al., 2011; Maniglia et al., 2020).

In particular, Maniglia and colleagues trained participants on peripheral collinear modulation and observed a reduction of critical space of crowding after training (Maniglia et al., 2011). Authors interpreted these results as supportive of the hypothesis of shared neural substrates, with reduced cortical inhibition after training contributing to the reduction of visual crowding.

However, the authors did not directly compare training gain and transfer gain to address the hypothesis.

Here, we used linear regression methods to test whether the learning gain observed in collinear inhibition is related to the transfer gain to crowding reduction. Surprisingly, results showed a significant, but inverse correlation between reduction of collinear inhibition (measured at the shortest separation of 2λ) and reduction of visual crowding, suggesting a less straightforward interaction between the two phenomena. We interpret this inverse correlation as possibly resulting from a lack of transfer when training is too specific to one of the sub-conditions. This aligns with previous findings in the lateral masking literature (Polat & Sagi, 1994) and with studies and models addressing learning and generalization (Hung & Seitz, 2014; Maniglia & Seitz, 2018; Xiao et al., 2008).

When learning is limited to a single separation (or, more precisely in the context of this paper, when one separation shows proportionally more learning effect than the others), it might disrupt the local chain of lateral interactions, thus compromising collinear facilitation for the untrained separations (or in this case, separations that show less learning effect) and preventing transfer (Polat & Sagi, 1994).

## Method

### Participants

Eight healthy vision participants took part in the original study. Details can be found in Maniglia et al. (2011).

### Apparatus and Procedure

Participants took part in a peripheral contrast detection training using a lateral masking configuration. Before and after training, participants underwent a visual crowding task to assess transfer of learning. Further details can be found in Maniglia et al. (2011).

### Data Analysis

To analyze the relationship between collinear modulation and crowding, we used Pearson's correlations and linear multiple regression gain model in R. The dependent variables of interest for the Pearson's correlations were crowding thresholds (separation between target and flankers leading to 79% correct identification (Whitney & Levi, 2011)) at pre- and post-test, separately.

The dependent variable for the multiple regression was crowding thresholds at post-test. To estimate crowding improvements, pre-test crowding thresholds were used as a covariate. This approach is considered more effective in capturing longitudinal changes as it controls for baseline performance and does not assume that the regression coefficient of pre versus post performance is 1.0 (Cohen, 2013). To investigate the relationship of crowding change and training, training threshold gains at the various flanker separations were added as covariates. Training thresholds were calculated as log[iso/ortho] flanker orientation ratios (Maniglia et al., 2011; Maniglia et al., 2015b; Shani & Sagi, 2005). Training gains were then calculated by subtracting post- from the pre-training thresholds.

## Results

### Correlation Between Lateral Inhibition and Critical Space of Crowding

**Figure 1.**
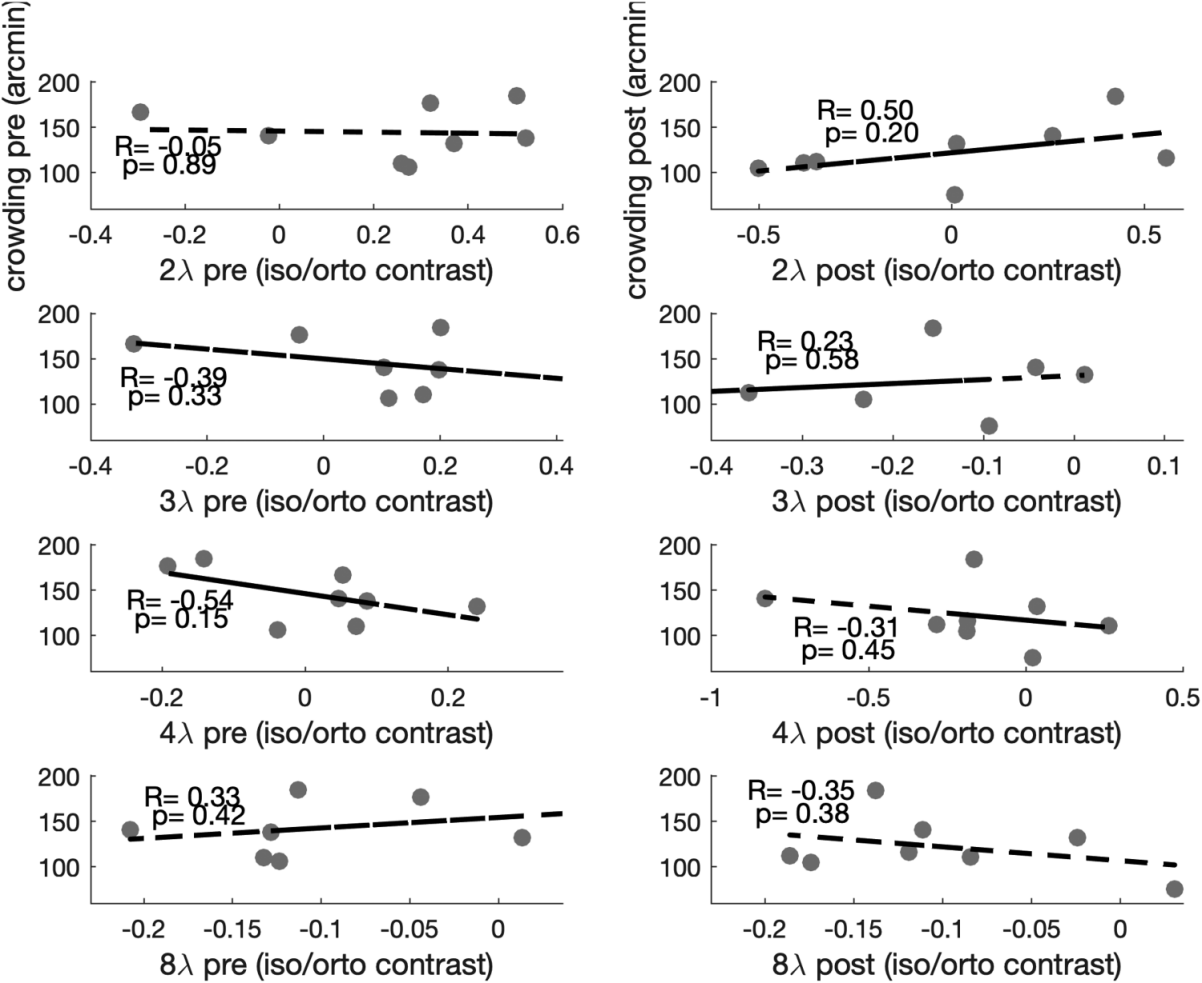
Within-participant correlations between crowding and collinear modulation. Larger values on the *y*-axis indicate worse crowding performance, whereas positive values on the *x*-axis indicate collinear inhibition (collinear contrast thresholds higher than orthogonal thresholds).

First, we looked at the within-participant correlations between contrast threshold ratios (iso/ortho), measured at different separations, and critical space of crowding. Results showed no significant correlation between collinear inhibition (2λ, 3λ, and 4λ) or facilitation (8λ) and crowding at baseline or after training (see Figure 1).

### Correlation Between Training Gain and Transfer to Crowding

**Figure 2.**
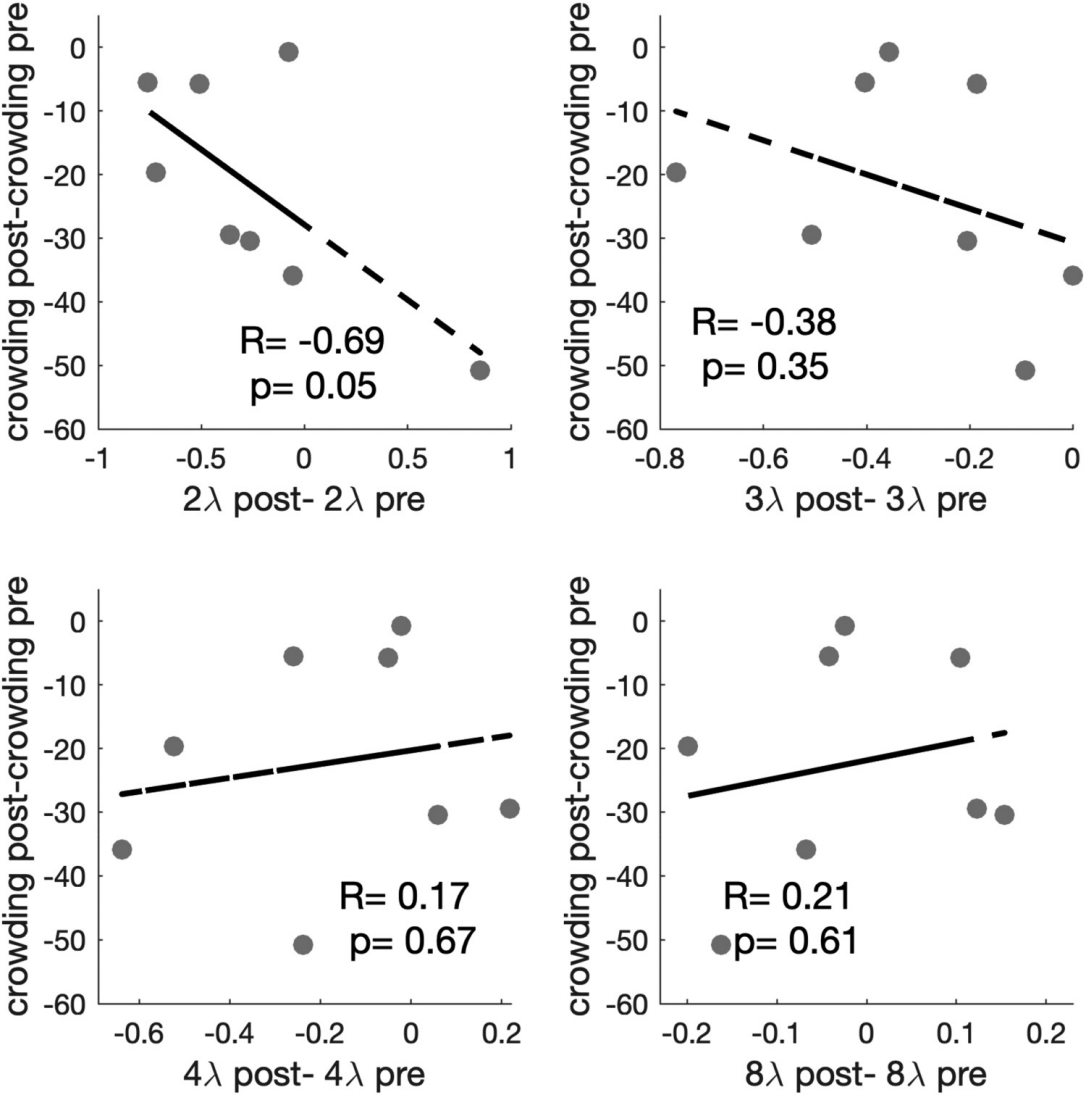
Within-participant correlations between changes in crowding and collinear modulation training. The *y*-axis represents the change in visual crowding (post-training minus the baseline performance), measured in arcminutes of the visual angle. The *x*-axis represents the change in contrast detection performance across different flanker separations (post-training the minus baseline performance).

Next, we looked at the correlation between training gain in both tasks (Figure 2). We found no significant correlations, except for the marginally significant test at 2λ.

We then compared linear regression models predicting crowding performance from training gain at each target-to-flanker separation (Table 1). This analysis found that the best model based on parsimony and model fit (Kenny, 1979) was Model 1, in which training gains at 2λ predicted transfer to crowding. Specifically, results show a negative coefficient of 2λ, indicating that an improvement at 2λ due to training (indicated by a negative threshold difference) leads to higher (worse) post-test crowding thresholds (indicating a smaller transfer gain).

**Table 1.** Regressed crowding change model comparisons. For each model, we present the relevant statistical information.

Parameter	Coefficient	Std. error	*t*-Value	*p*
Model 0 (Crowding Post ∼ Crowding Pre)
Intercept	−8.21	35.47	−0.23	.82
Crowding Pre	0.9	0.24	3.74	.009
*F*(1,6)= 13.98, *p* = .01		Adjusted *R*^2^ = .65		
AICc = 79.22				
BIC = 73.46				
Model 1 (Crowding Post ∼ Crowding Pre + 2λ)
Intercept	−65.04	33.57	−1.94	.11
Crowding Pre	1.24	0.22	5.72	.002
2λ	−32.04	12.37	−2.58	.048
*F*(2,5)= 16.99, *p* = .006		Adjusted *R*^2^ = .82		
AICc = 81.75				
BIC = 68.74				
Model 2 (Crowding Post ∼ Crowding Pre + 3λ)				
Intercept	−27.58	43.17	−0.64	.55
Crowding Pre	0.98	0.26	3.7	.014
3λ	−26.03	31.18	−0.83	.44
*F*(2,5) = 6.98, *p* = .036		Adjusted *R*^2^ = .63		
AICc = 87.51				
BIC = 74.49				
Model 3 (Crowding Post ∼ Crowding Pre + 4λ)
Intercept	−9.67	38.69	−0.25	.81
Crowding Pre	0.92	0.27	3.43	.018
4λ	8.96	27.02	0.33	.75
*F*(2,5)= 6.01, *p* = .046		Adjusted *R*^2^ = .58		
AICc = 88.38				
BIC = 75.36				
Model 4 (Crowding Post ∼ Crowding Pre + 8λ)
Intercept	−13.47	40.83	−0.33	.75
Crowding Pre	0.94	0.28	3.35	.02
8λ	23.31	62.33	0.37	.72
*F*(2,5)= 6.06, *p* = .046		Adjusted *R*^2^ = .59		
AICc =88.33				
BIC = 75.32				

Looking at individual lambda gain as a predictor of learning transfer to crowding, the model that includes only 2λ as a predictor has the lowest AICc (small-sample corrected Akaike Information Criterion) aside from the model using only pre-test crowding scores, and the lowest BIC (Bayesian Information Criterion). This suggests that the 2λ-only model provides the best balance between fit and parsimony. In contrast, the more complex model including all lambda separations as predictors was excluded due to high variance inflation factors (>7) and overparameterization relative to the sample size for a reliable AICc calculation, as the number of predictors (6) approached the number of participants (8).

## Discussion

The relationship between visual crowding and lateral masking has been discussed in vision science for decades. On one hand, several researchers propose that the two phenomena are independent (Chakravarthi & Cavanagh, 2009; Pelli, 2008; Pelli et al., 2004), on the other hand, others believe the two to be related (Chung et al., 2001; Doron et al., 2015; Petrov et al., 2006; Polat & Sagi, 1993). The latter suggests that crowding and lateral masking share some basic features, hinting at common neural substrates. These are usually identified in the long-range horizontal connections of V1, linking units with similar orientation tuning (Bringuier et al., 1999; Cass & Spehar, 2005; Gilbert & Wiesel, 1985, 1989; Grinvald et al., 1994; Polat et al., 1998). This hypothesis is supported by electrophysiology (Polat et al., 1998) and neuroimaging (Millin et al., 2014) evidence for lateral masking and crowding, respectively (but see (Chicherov et al., 2014; Henry & Kohn, 2022; Motter, 2006) for a higher-level hypothesis on the neural substrates of crowding). Additionally, both phenomena appear modulated by feedback from extrastriate areas (Hupé et al., 1998; Levi, 2008; Maniglia et al., 2019)

However, these two phenomena differ in several aspects. In crowding, the target does not become less “visible,” as in collinear inhibition (Polat & Sagi, 2007), but rather unidentifiable (Levi, 2008). Electrophysiological evidence further suggests that crowding may occur in extrastriate regions, such as V4 (Chicherov et al., 2014).

Here, we reanalyzed data from Maniglia et al. (2011), a study showing that training contrast detection with lateral masking led to reduction of crowding. Authors interpreted this as supporting evidence of (at least partially) shared neural substrates. However, the authors did not directly test the relationship between learning in the collinear task and transfer to crowding. Here, we offer further evidence to support this relationship, although in a less intuitive way: we found a significant, but inverse correlation between post-training reduction of collinear inhibition and critical space of crowding, so that larger reductions of collinear inhibition (at short separations) led to smaller reductions of crowding.

This relationship is significant for the shortest flanker separations (2λ and 3λ), where collinear inhibition is systematically observed (see Figure 1), but not for larger separations. This is consistent with the hypothesis that crowding is related to collinear inhibition, but not to collinear facilitation, which previous authors suggest depending upon different mechanisms. Additionally, this is consistent with evidence suggesting that training on lateral masking leads mostly to reduction of inhibition at short flankers’ separations rather than increase in facilitation at larger separations. Lev and Polat (2011) propose that the relationship between crowding and lateral interactions is characterized by the perceptual field (PF), the perceptual correspondent of the physiological receptive field. In this framework, collinear inhibition emerges as a within-PF effect, with facilitation being a between PFs effect. Similarly, crowding might depend on flankers located within the same perceptual region of integration than the target, so that the configuration is grouped into a whole (Manassi et al., 2013). Indeed, both crowding and PF size increase with eccentricity (Bouma, 1970; Lev & Polat, 2011) while being negligible or absent in the fovea (Levi et al., 2002).

However, it is important to note that while the lateral masking literature [alongside other evidence, for example, Henry & Kohn (2020); Millin et al. (2014); Nandy and Tjan (2012); Shin et al. (2017)] supports a “V1-centric” interpretation of crowding, alternative explanations involving higher-level visual areas are equally prominent. In particular, evidence from neurophysiology (Motter, 2006, 2018), neuroimaging (He et al., 2019) and psychophysics suggests that multiple brain areas, including extrastriate regions (Anderson et al., 2012; Bi et al., 2009; Tyler & Likova, 2007) and beyond (e.g., Louie et al. , 2007), contribute to crowding, along with higher-level mechanisms such as visual attention (Fang & He, 2008). Thus, the current results only partially address a more complex phenomenon. As Whitney and Levi (2011) noted, “any single visual area could explain this range of effects is therefore tenuous, at best.”

Similarly, while we, along with other authors, frame our results in the context of the relationship between lateral interactions and crowding, an alternative explanation is that improvements in contrast thresholds per se had an alleviating effect on crowding. Indeed, Rodriguez and Granger (Rodriguez & Granger, 2021) posit that differences in contrast can explain key characteristics of crowding, and several studies have shown a reduction in the crowding effect for low-contrast stimuli (Moshkovitz et al., 2022; Pascal & Abadi, 1995; Simmers et al., 1999).

What may seem counterintuitive at first is the inverse relationship between collinear inhibition and crowding, so that those who improved the most after training at the shortest separations (the more inhibitory condition) showed the smallest amount of transfer to crowding.

**Figure 3.**
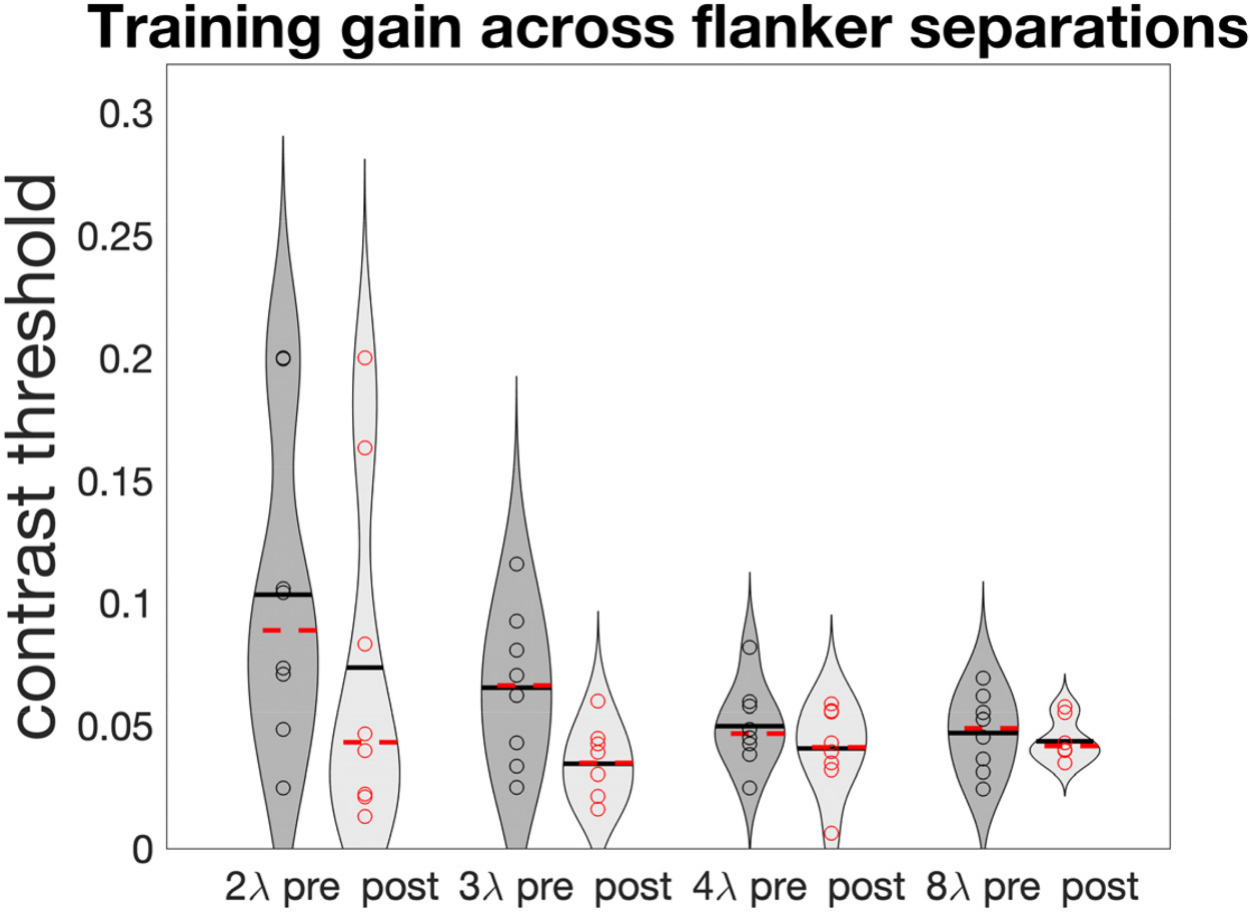
Pre- versus post-contrast thresholds (iso orientation) across lambdas.

A possible explanation is that too-specific learning limits transfer: Indeed, when looking at training data, we observe that most of the learning took place at the shortest separations (Figure 3), with 2λ and 3λ showing the largest improvement (pre vs. post *t*-tests showing significant learning at 3λ (*t*_7_ = 3.33, *p* = .013, and, removing one outlier in the opposite direction of the overall trend, at 2λ as well, *t*_6_ = 2.93, *p* = .026). Those are the separations that learned the most and possibly gatekept transfer. This result is consistent with previous evidence suggesting that lateral masking training results mostly in reduction of inhibition (i.e., improvements at short separations) rather than increase in facilitation (Maniglia et al., 2011, 2018; Polat & Sagi, 1994). Still, the progressively reversing correlation between gains as separations increase (Figure 2) hints at a more complex relationship between collinear inhibition, facilitation, and crowding.

Doron et al. (2015) characterized the relationship between crowding and lateral masking in children, reporting a progressive reduction of crowding as collinear facilitation emerges. Here, we show that training on collinear inhibition affects visual crowding. However, several methodological differences between the two studies prevents us from comparing the results more in depth, and possibly explain why we did not observe a correlation between crowding and collinear inhibition before or after training, despite their learning gain being related. This is also consistent with evidence suggesting that performance across different visual tests does not necessarily correlate within the same individuals (Cappe et al., 2014).

To conclude, by reanalyzing data from Maniglia et al. (2011), we confirmed the original hypothesis of the authors, that is, training on collinear inhibition leads to improvements in visual crowding, albeit in the opposite direction: larger training gain led to smaller transfer gain. We interpret this as indication that, to induce transfer of learning to a visual function that shares common substrates with the trained task, training effects must be distributed across separations rather than concentrated on a subset of conditions. This interpretation is consistent with both previous training studies on lateral interaction (Polat and Sagi, 1994) and current models of PL explaining generalization and transfer of learning (Maniglia and Seitz, 2018; Xiao et al., 2008). If unequal learning across separations does gatekeep transfer of learning, future training studies using lateral masking might implement algorithms that adjust the number of trials of the difficulty of the training to ensure equal or at least similar learning curves across lambdas.

An important limitation of this study is the small number of participants, which invites caution when interpreting correlation and discussing statistical significance. While relatively common in training studies (Ball & Sekuler, 1987; Fiorentini & Berardi, 1981; Rodán et al., 2022), and specifically with lateral masking (Polat & Sagi, 1994), it still represents a major limitation to the present report's conclusions. Another limitation of this study lies in the design of Maniglia et al. (2011), which employed a yes–no paradigm. This approach is known in the collinear modulation literature to be prone to filling-in and false alarms (FAs) (Polat & Sagi, 2007). While the original paper found that the training effect did not impact the rate of FAs, suggesting that the improvement was perceptual rather than a change in response criterion, future studies measuring collinear modulation should rely on more robust 2AFC procedures.
